# Sensitive Cross-Linked SnO_2_:NiO Networks for MEMS Compatible Ethanol Gas Sensors

**DOI:** 10.1186/s11671-020-3269-3

**Published:** 2020-02-05

**Authors:** Weiguang Tong, Ying Wang, Yuzhi Bian, Anqi Wang, Ning Han, Yunfa Chen

**Affiliations:** 1 State Key Laboratory of Multiphase Complex Systems, Institute of Process Engineering, Chinese Academy of Sciences, Beijing, 100190 China; 2 State Key Laboratory of Chemical Resource Engineering, Beijing Key Laboratory of Environmentally Harmful Chemicals Analysis, Beijing University of Chemical Technology, Beijing, 100029 China; 3 Department of Physics, School of Science, Beijing Jiaotong University, Beijing, 100044 China; 4 Center of Materials Science and Optoelectronics Engineering, University of Chinese Academy of Sciences, No. 19A Yuquan Road, Beijing, 100049 China

**Keywords:** Cross-linked SnO_2_:NiO network, Self-assembly, Gas sensor, MEMS compatible, Ethanol detection

## Abstract

Nowadays, it is still technologically challenging to prepare highly sensitive sensing films using microelectrical mechanical system (MEMS) compatible methods for miniaturized sensors with low power consumption and high yield. Here, sensitive cross-linked SnO_2_:NiO networks were successfully fabricated by sputtering SnO_2_:NiO target onto the etched self-assembled triangle polystyrene (PS) microsphere arrays and then ultrasonically removing the PS microsphere templates in acetone. The optimum line width (~ 600 nm) and film thickness (~ 50 nm) of SnO_2_:NiO networks were obtained by varying the plasma etching time and the sputtering time. Then, thermal annealing at 500 °C in H_2_ was implemented to activate and reorganize the as-deposited amorphous SnO_2_:NiO thin films. Compared with continuous SnO_2_:NiO thin film counterparts, these cross-linked films show the highest response of ~ 9 to 50 ppm ethanol, low detection limits (< 5 ppm) at 300 °C, and also high selectivity against NO_2_, SO_2_, NH_3_, C_7_H_8_, and acetone. The gas-sensing enhancement could be mainly attributed to the creating of more active adsorption sites by increased stepped surface in cross-linked SnO_2_:NiO network. Furthermore, this method is MEMS compatible and of generality to effectively fabricate other cross-linked sensing films, showing the promising potency in the production of low energy consumption and wafer-scale MEMS gas sensors.

## Introduction

Volatile organic compound (VOC) sensing has been attracting more and more attention due to its significance in environment monitoring, production safety, and human health care [1–5]. As one of the most common and important VOCs, ethanol is the main component to be detected in drunk driving test. The resistive ethanol sensors used semiconducting metal oxides (MOS) as sensing materials are popular due to their advantages, such as cheap, nontoxic, stable, simple processing, and higher sensitivity performance [6–8]. Typically, various nanostructured MOS including nanowires, nanoplates, hollow spheres, and heterostructures can greatly enhance the diffusion of analyte gases and facilitate the charge transport, leading to high sensitivity and fast sensing-recovery process [9–18]. However, most of the reported sensors are fabricated by drop-coating or screen printing the nanostructured MOS solution onto ceramic tubes or plates, which results in large sensor-to-sensor variations, large size, and high power consumption of 200–1000 mW [7, 19–23]. Another challenge is the agglomeration between nanostructures by strong van der Waals attractions, which leads to decreased sensitivity and low uniformity [24]. To avoid these disadvantages, substrates with low energy dissipation and new technology of sensing material integration are required before their practical commercial applications.

Nowadays, microelectrical mechanical system (MEMS) sensors developed with microfabrication methods can accomplish the device miniaturization, low power consumption, good consistency, and wafer-scale device production. Microheaters allow for high sensing temperatures to be reached with low input power by the design of a small and suspend heater area thermally isolated from the bulk substrate [25–28]. Various traditional MOS thin films can be integrated on the microheaters also by MEMS techniques such as spraying, thermal evaporation, sputtering, physical vapor deposition (PVD), atomic layer deposition (ALD), chemical vapor deposition (CVD), etc. [29–32]. The collaboration of different MEMS sensors can facilitate the development of array technology to detect gases in complex contexts, which is the prototype of electronic nose (e-nose) [33–35]. Despite of these advantages, challenges still exist in the following three aspects. First, the traditional MOS thin films by MEMS techniques often show poor sensitivity to target gases due to the compact surface structure and low crystallinity. For example, Kang et al. reported a sputtered Pt-doped SnO_2_ thin film on microheater with a sensitivity of less than 4 to 25 ppm toluene at 450 °C [29]. All the sputtered SnO_2_:NiO thin films in our previous research showed low sensor response of < 2 to 5 ppm NO_2_ at 200 °C before incorporating the self-assembled Au nanoparticle array [25]. Second, some researchers have tried to integrate high-performance MOS nanomaterials onto microheaters, but it is difficult to control and cast the slurry-based MOS nanomaterials onto the suspending heating area of microheaters. Several groups have reported the fabrication of nanomaterial-based MEMS sensors via ink-jet printing, polymeric mask centrifugation, and dip pen nanolithography (DPN) methods [12, 36–39]. However, the low yield and large device-to-device deviation hampers the sensor fabrication in a large scale. Third, it is also complicated to improve the adhesion between microheater and sensing nanomaterials in order to get stable parameters especially at high temperature > 350 °C. In our previous research, we found that the mix of dielectric glass dust with hollow SnO_2_ nanospheres was required to improve adhesion between SnO_2_ sensing membrane and MEMS microheater, resulting in decreased sensing performance and low stability [24]. Fabricating sensing films with high sensitivity using MEMS compatible methods is an urgent goal.

Design of nanostructures with large surface area in the traditional MEMS thin films is the key strategy, because the sensor sensitivity is positively attributed to surface adsorption of the sensing film. A low enthalpy of the adsorbed phase is often expected when a gaseous molecule is adsorbed on the sensing film with lots of stepped and kinked surfaces [9]. Therefore, sensing materials like three-dimensional pore arrays and cross-inked networks tend to adsorb more gaseous molecules and realize sensitive gas-sensing [40–42]. The use of sacrificial templates such as self-assembled polystyrene (PS) spheres array is one of the effective, relatively cheaper, and MEMS compatible ways to form large-scale uniform step-rich morphology on sputtered MOS thin films [9, 42]. And the size, period, and shape of the PS nanostructures can be controlled by further plasma etching. For example, triangle array or cross-linked network can be formed depending on the plasma etching time of PS spheres through the same processes: (i) self-assemble PS spheres, (ii) plasma etching of PS spheres, (iii) deposit MOS thin film, and (iv) remove PS spheres. Apart from creating more active adsorption sites, forming heterostructure to improve the sensing performance of MOS-based gas sensors has been intensively studied, which is a low cost, environmental-friendly, and easy-to-implement method [25, 43–48]. The sputtering target can be designed by mixing two or more MOS elements, such as SnO_2_/NiO, SnO_2_/ZnO, SnO_2_/WO_3_, etc. Besides, the component and element ratio of hybrid sensing films can be flexibly controlled by co-sputtering two targets at different sputtering power. Considering the easy accessibility of nanostructured morphology and heterostructures by templates and sputtering techniques, new type of MEMS sensors with high sensor response can be put forward.

In this work, by the MEMS compatible colloidal-monolayer-based method, a series of cross-linked SnO_2_/NiO networks were prepared with different periodic structures. The self-assembled close-packed PS microsphere (diameter ~ 1 μm) arrays were explored as templates, the size of which could be in wafer-scale when assembled in Langmuir-Blodgett (LB) troughs. To fabricate cross-linked SnO_2_/NiO networks, the ball-to-ball gaps of PS microspheres templates were tuned by plasma etching for different time (0–30 min), and then SnO_2_/NiO thin layers were sputtered onto the etched templates followed by removing PS microspheres. Compared with continuous SnO_2_/NiO films, the prepared heterostructured cross-linked networks exhibited a significantly enhanced response to ethanol vapor (~ 9 to 50 ppm) and a wide working temperature range (300–375 °C). A detection limit of 5 ppm was realized at a working temperature of 300 °C. These results demonstrate that the creation of stepped surfaces in cross-linked structure can effectively enhance the gas-sensing of traditional sputtered thin films. As a proof of concept, this work provides a flexible strategy for designing other cross-linked thin films for practical MEMS gas sensors and sensor arrays.

## Materials and Methods

### Fabrication of PS Microspheres Array Template

Clean substrates with 300-nm-thick Si_3_N_4_ on both sides of p-type Si (Jingyifang Electronics Co., Ltd.) were used and cut into two sizes of small pieces (1 cm × 1 cm and 2 cm × 4 cm). The use of Si_3_N_4_ substrate instead of SiO_2_ is necessary, because Si_3_N_4_ can serve as the mask when fabricating the hollow cavity by wet etching technique in KOH solution, as shown in Figure S1 in our previous work [25]. Polystyrene (PS) microspheres (250 mg/ml, BIOPEONY) with 1.0 μm in diameter were used after diluted by 50% in ethanol (99.99%, Beijing Chemical Reagent Co. Ltd.). Cetyltrimethyl ammonium bromide (CTAB, ≥ 99%, SIGMA) was used to control the surface wettability.

**Fig. 1:**
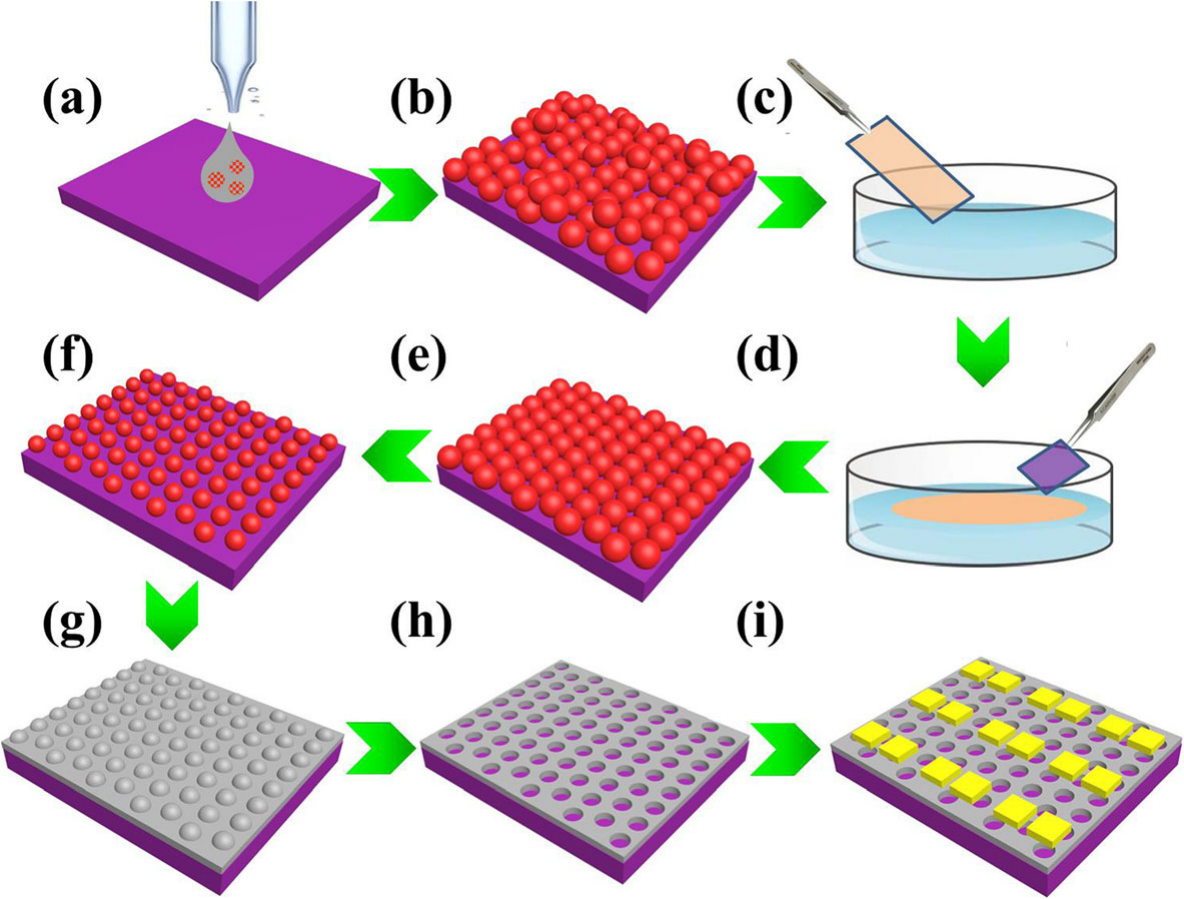
Schematic illustration of the fabrication processes for cross-linked network based gas sensors. **a** Drop PS microspheres solution onto a 2 cm × 4 cm Si_3_N_4_ substrate. **b** PS microspheres self-assemble into an irregular monolayer. **c** Insert the above Si_3_N_4_ substrate into deionized water. **d** PS microspheres reassemble into a close-packed regular array floating at the air/water surface. **e** Another 1 cm × 1 cm Si_3_N_4_ substrate was used to carefully pick up the two-dimensional array. **f** Plasma etching was executed to control the size of PS microspheres. **g** Deposit the SnO_2_/NiO thin film by sputtering technique. **h** Remove the PS microspheres to form a cross-linked SnO_2_/NiO network. **i** Deposit the gold electrodes array

First of all, all the Si_3_N_4_ substrates and water containers were treated by a radio frequency plasma source (YZD08-5C, Saiaote Technology Co. Ltd.) for 30 s at a power of 200 W to create hydrophilic surfaces. Two drops of diluted PS microspheres solution were cast onto a 2 cm × 4 cm Si_3_N_4_ substrate (Fig. 1a). As the ethanol evaporated, PS microspheres self-assembled into an irregular monolayer (Fig. 1b). Then, a 20 μl 5 g/L CTAB solution was added to 100 ml deionized water in a glass container to modify the surface tension of water. As the above Si_3_N_4_ substrate slid slowly into the water in the flume, the irregular PS microspheres reassembled into a close-packed PS microspheres array floating on the water surface, as shown in Fig. 1c, d. Another clean 1 cm × 1 cm Si_3_N_4_ substrate was then inserted to carefully pick up the close-packed PS microspheres array (Fig. 1e). Finally, the size of PS microspheres was tuned by changing the plasma etching time at a constant input power of 200 W (Fig. 1f).

### Fabrication of Cross-Linked SnO_2_/NiO Networks

The SnO_2_/NiO (NiO 1%, SnO_2_ 99%) MOS target material for magnetron sputtering (Kurt J. Lesker, LAB 18) was purchased from Jiangxi Ketai New Material Co. Ltd. Thin SnO_2_/NiO films with the thickness of 20 nm, 50 nm, and 100 nm on the etched PS microspheres array templates were obtained by sputtering the same target for 430 s, 1075 s, and 2150 s at a power of 80 W (Fig. 1g). Cross-linked SnO_2_/NiO networks were then formed after removing the PS microspheres in acetone, as shown in Fig. 1h. As most of the as-deposited thin films by sputtering are non-crystalline, the network films were post-annealed at high temperature of 500 °C in reduction condition (5% H_2_, 95% Ar) for 2 h.

### Characterization of Cross-Linked SnO_2_/NiO Networks

The overall structure and morphologies of PS microspheres and cross-linked sensing networks were investigated by a scanning electron microscope (SEM, JEOL JSM-6700F) operated between 10 and 20 kV. The crystalline phase of sensing films was studied by small-angle X-ray scattering (SAXS, Panalytical X’pert Pro) with a Cu Kα radiation source (wave length = 1.5406 Å) at *2θ* angles ranging from 20° to 80°. In addition, the elements and chemical states on surface of the films were investigated by X-ray photoelectron spectroscopy (XPS, Thermo Fisher ESCALAB 250Xi) with monochromatic Al Kα radiation (*hν* = 1486.6 eV; *h* is Planck’s constant and *ν* is frequency). All binding energies were calibrated with respect to the signal adventitious carbon C1s peak with a binding of 284.7 eV. The fitted peaks in the XPS spectra were separated using the XPSPeak 4.1 software.

### Device Fabrication and Measurement

**Fig. 2:**
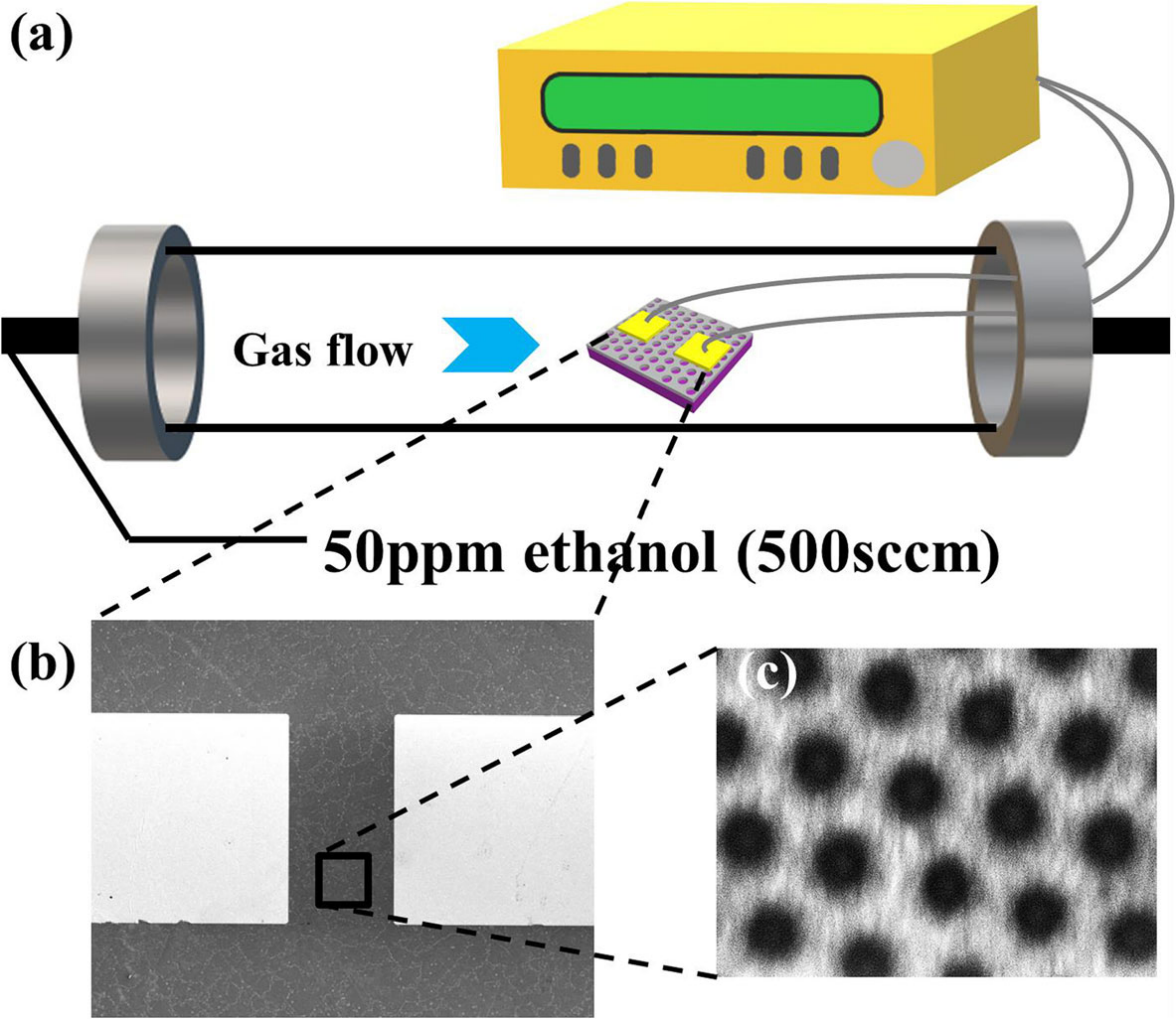
**a** Schematic diagram of the homemade gas-sensing instrument. **b** SEM image of a measured device. **c** Magnified SEM image showing the cross-linked SnO_2_/NiO sensing network

Gold electrodes (Cr/Au∼10/80 nm) were then fabricated on the cross-linked network by lithography (SUSS MicroTec, MA6) and electron beam evaporator technique (OHMIKER-50B), as shown in Fig. 1i. Wafer-scale cross-linked MOS gas sensors can also be fabricated by subsequent photolithography and etching techniques, according to the technological process in our previous paper [25]. For gas response, the gas-sensing property of our prepared SnO_2_/NiO network sensors in Fig. 1i was measured in a homemade dynamic instrument, as shown in Fig. 2a. In detail, the probes of Pt wires on the instrument were connected with gold electrodes of sensors by an intermediate ceramic chip. Micro-sized gold electrodes on sensors were firstly connected with the gold pads (Ti/Au 10/200 nm) on the ceramic chip by a wire-bonding machine (aluminum wires, Shenzhen Shunyu Automatic Equipment Co. LTD., WL2046). The Pt wire probes were then electrically contacted with the gold pads on the ceramic chip by silver paste (Wuhan Youle Optoelectronics Technology Co., LTD.). The current-time curves were measured using a sourcemeter at a constant bias of 5 V (Keithley, 2620B). All the used gases were purchased from Beijing Hua Yuan Gas Chemical Industry Co., Ltd. To prepare a targeted gas with a specific concentration, the synthetic air and standard gas (ethanol, NO_2_, NH_3_, and other gases in synthetic air) were mixed at a certain ratio controlled by two digital mass flow controllers (Tianjin Zhonghuan Experimental Furnace Co. LTD.) at a total flow rate of 500 ml min^−1^. The testing temperature was varied from 200 to 400 °C. The response of the sensors was calculated by the resistance ratio between in the air (*R*_a_) and in the target gas (*R*_g_), (*R*_g_/*R*_a_-1) for NO_2_ and (*R*_a_/*R*_g_-1) for other gases.

## Results and Discussion

### Morphological, Component, and Chemical States Characterization

Figure 2b shows the SEM image of a typical device, characterized after all gas-sensing measurements. To make the cross-linked structure more prominent, the source and drain electrodes were separated by 100 μm, so that an amount of 80 holes can be included along the channel. The fine structure with a resistance of 10 GΩ also provides an adequate base line for gas-sensing tests. The 10 nm/80-nm-thick Cr/Au pads were designed with the size of 200 μm × 200 μm, large enough for wire-bonding by silver paste. Figure 2c shows the magnified SEM image of the area framed by the rectangle in Fig. 2b. It is clear that the sensing film in the channel is composed of cross-linked SnO_2_/NiO networks.

**Fig. 3:**
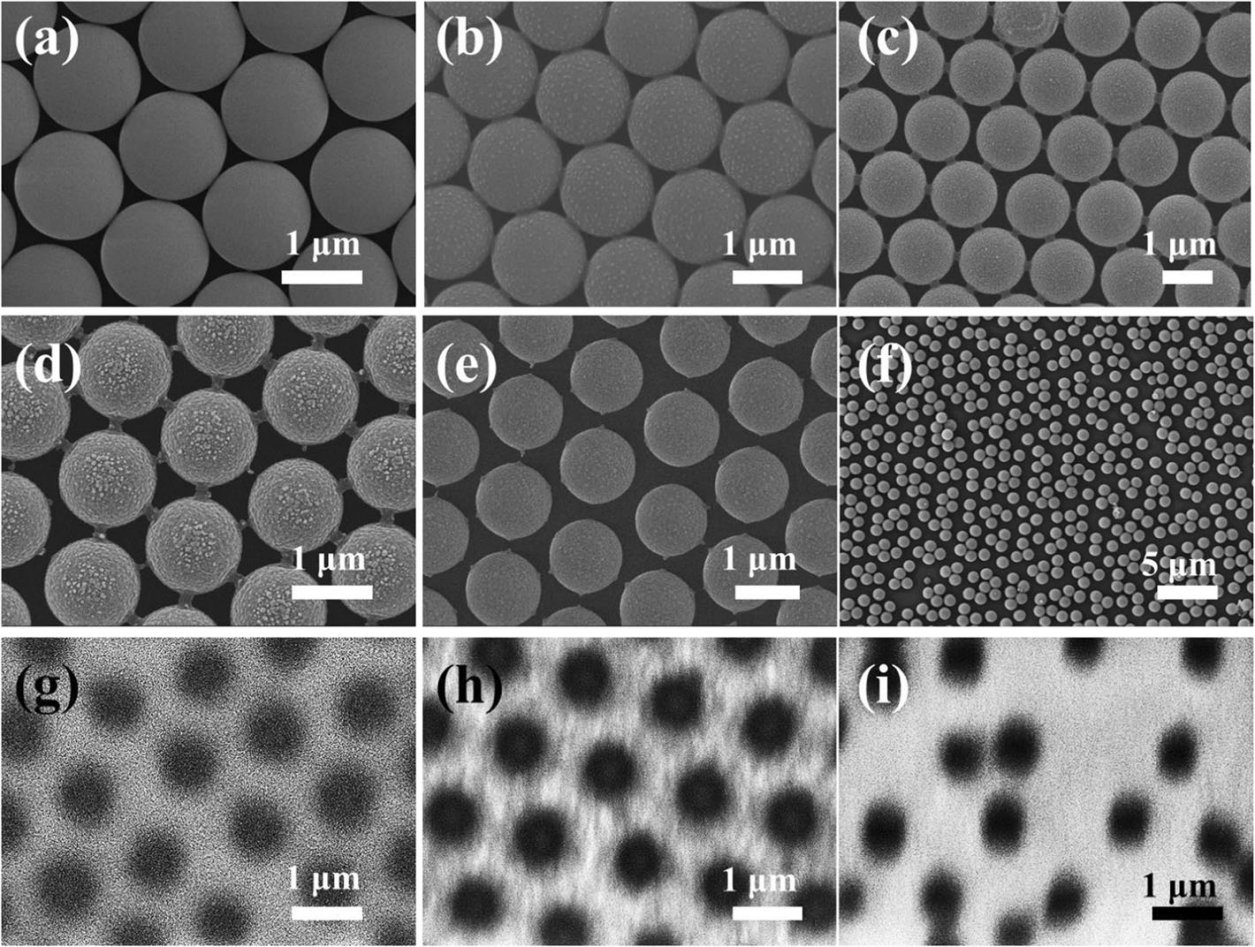
PS microspheres templates etched for 0 min (**a**), 5 min (**b**), 10 min (**c**), 15 min (**d**), 20 min (**e**), and 30 min (**f**). Displacement was observed for PS microspheres etched for 30 min, resulting in a disorder PS array. **g**–**i** The corresponding cross-linked networks after removing the PS microspheres templates etching for 15 min, 20 min, and 30 min. Networks could not be formed for templates etched less than 15 min, because the gap between two adjacent PS microspheres were too small

The line width and the diameter of holes in cross-linked SnO_2_/NiO networks were tuned by changing the plasma etching process. Figure 3a demonstrates the SEM image of an ordered PS microspheres superlattice in hexangular close-packed structure, which was prepared without plasma etching. As the time of etching treatment increased, the size of PS microspheres decreased obviously, as shown in Fig. 3b–e. Adjacent PS microspheres began to separate after plasma etching for 10 min, leaving narrow interconnecting wires which were attributed to the glass transition of PS microspheres. Only discrete triangular SnO_2_/NiO patterns can be formed if we use this type of PS microspheres template, in which no conductive path exists. In Fig. 3d, the interconnecting wires began to break as the plasma etching time increased to 15 min, in which case the corresponding cross-linked SnO_2_/NiO networks began to form. After 20 min of etching, the interconnecting wires around PS microspheres disappeared, as shown in Fig. 3e. Displacements were observed in PS microspheres array etched for 30 min due to the high power accumulation, which leads to a disorder PS array in Fig. 3f. Figure 3g–i shows the corresponding SnO_2_/NiO networks fabricated by the PS microspheres templates etched for 15 min, 20 min, and 30 min. The line widths for 15 min and 20 min etching templates are 400 nm and 500 nm, respectively. The SnO_2_/NiO network fabricated by 30 min etching templates is also disordered, as shown in Fig. 3i.

**Fig. 4:**
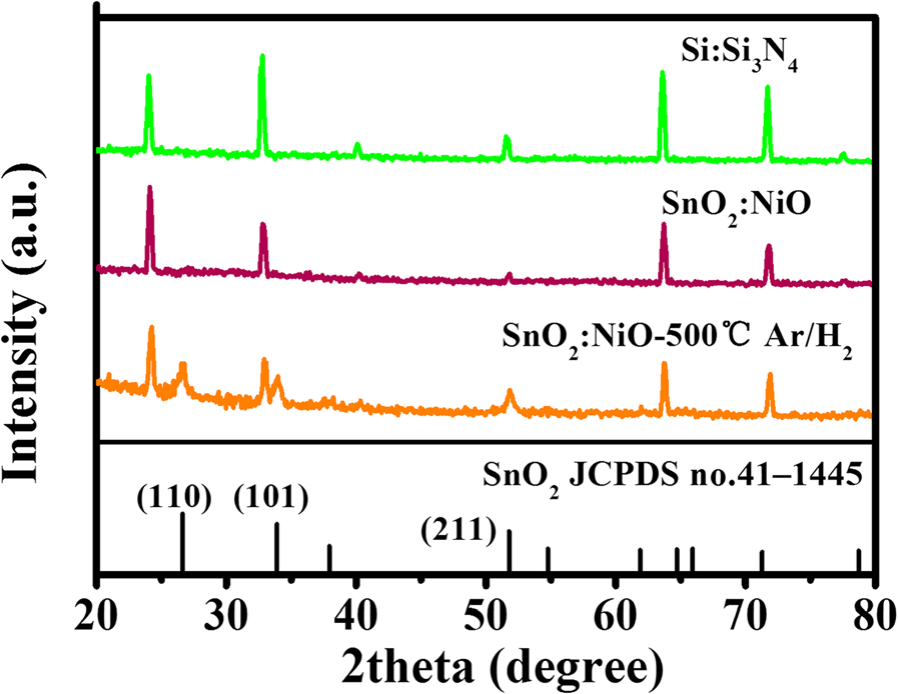
The SAXS characterization of the Si/Si_3_N_4_ substrate, the as-deposited SnO_2_:NiO film, and the SnO_2_:NiO film annealed at 500 °C

Most of the thin films deposited by sputtering, evaporation, CVD, PVD, or ALD techniques require a post-annealing process to reorganize and stabilize the original non-crystalline structure [25, 29, 30]. Thus, the cross-linked networks were post-annealed at high temperature of 500 °C in H_2_ for 2 h. The change of grain size and surface roughness were hard to distinguish due to the poor conductivity of SnO_2_/NiO for SEM characterization, whereas the SAXS patterns shows more details of the crystallinity in Fig. 4. The data of Si/Si_3_N_4_ substrate was included to deduct the impact of the background. The peaks in the SAXS pattern of the Si:Si_3_N_4_ substrate are attributed to Si_3_N_4_. (PDF 33-1160). Clearly, there is no obvious peaks appeared in as-deposited SnO_2_:NiO films indicating the amorphous structure. After activated by annealing in H_2_, obvious peaks were observed at 51.7°, 33.9°, and 26.6° corresponding to (211), (101), and (110) (JCPDS File no.41–1445), which indicated the formation of rutile SnO_2_. No characteristic peak of NiO was observed because of the little proportion.

**Fig. 5:**
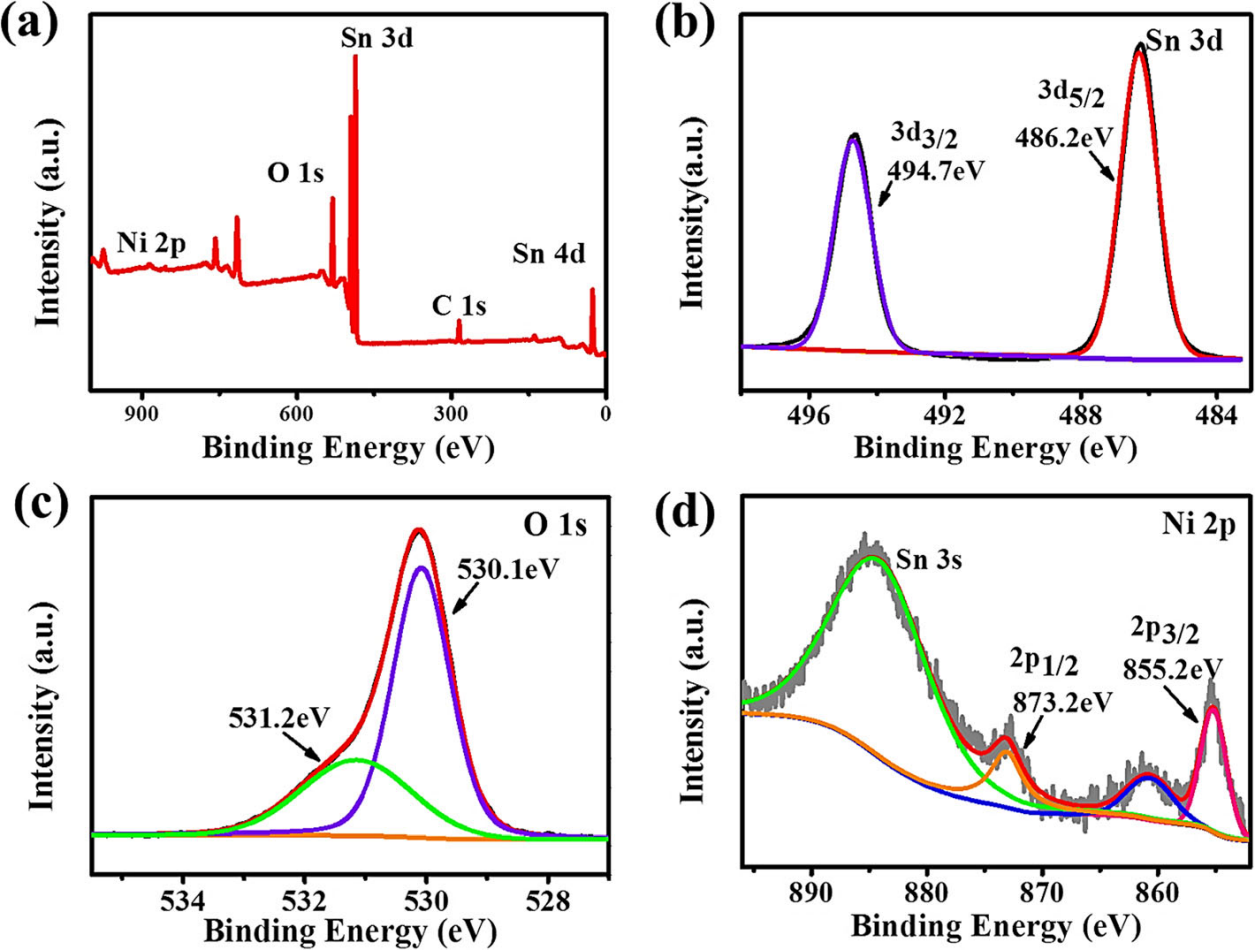
XPS spectra of **a** full spectrum, **b** Sn 3d, **c** O 1s, and **d** Ni 2p core-level spectra of annealed SnO_2_:NiO networks

For ethanol detection, gas sensing is based on the oxidation–reduction reaction of adsorbed ethanol on the surface of MOS, which leads to an abrupt conductance change in the sensing materials. Thus, the sensitivity is highly influenced by the surface elemental compositions and chemical states of annealed SnO_2_/NiO networks. Figure 5 shows the results of XPS analysis, in which the binding energies were calibrated by referencing the C 1s peak (284.8 eV) to reduce the sample charge effect. The full spectrum in Fig. 5a indicates the presence of Sn, O, and Ni in the SnO_2_:NiO composites. In Fig. 5b, two symmetric doublet peaks were observed centered at 486.2 eV (Sn 3d_5/2_) and 494.7 eV (Sn 3d_3/2_) with a spin-orbit splitting of 8.5 eV, indicating the presence of Sn in an oxidation state of + 4. Figure 5c shows that the surface oxygen species can be deconvoluted into two Gaussian component peaks centered at 530.1 and 531.2 eV, which are respectively corresponding to the lattice oxygen (O_latt_) and O^2−^ species. Given that the ethanol sensing performance is closely related to the O^2−^ ion, the high percentage of O^2−^ (~ 33.3%) might indicate lots of active adsorption sites in cross-linked SnO_2_/NiO networks. Distinct Ni 2p peaks in Fig. 5d located at 855.2 eV and 873.2 eV corresponding to Ni 2p_3/2_ and Ni 2p_1/2_ were observed, indicating the existing of Ni in the sensing composites in a valence state of 2+. This ratio of 1% between NiO and SnO_2_ has been optimized by balancing two aspects: the formation of effective p-n heterojunction and an adequate baseline of resistance, which has been discussed in detail in our previous work [25].

### Gas-Sensing Performance

**Fig. 6:**
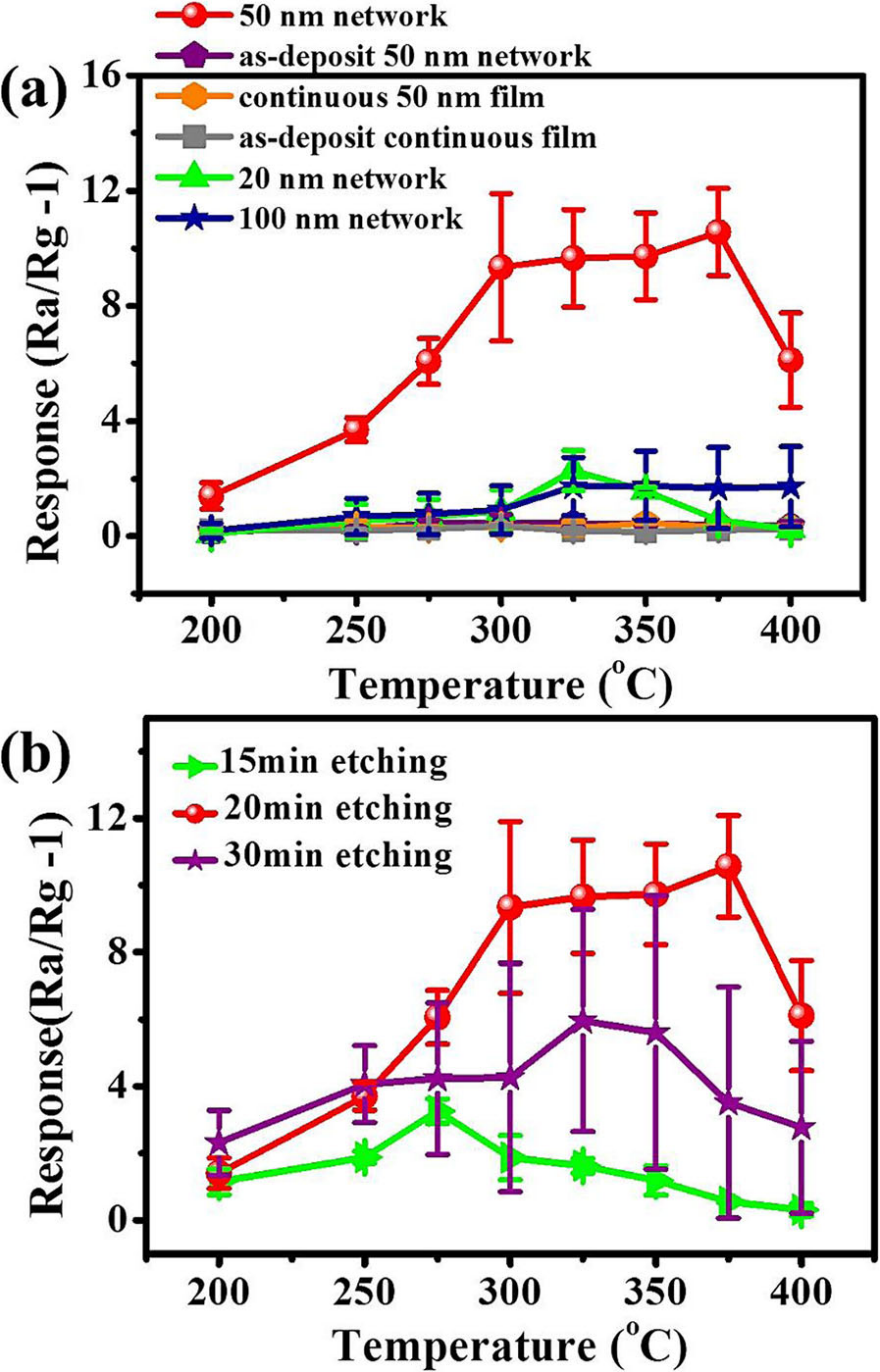
Sensor responses of various samples towards 50 ppm ethanol vapor. **a** Gas responses of the six types of sensors, based on annealed 50-nm-thick SnO_2_:NiO network as-deposit 50-nm-thick SnO_2_:NiO network, annealed continuous 50-nm-thick SnO_2_:NiO film, as-deposit continuous 50-nm-thick SnO_2_:NiO film, annealed 20-nm-thick SnO_2_:NiO network, and annealed 100-nm-thick SnO_2_:NiO network, respectively. **b** Gas responses of the sensors fabricated at different plasma etching time

Gas-sensing tests to 50 ppm ethanol were carried out for sensors based on films with different structural parameters, such as annealing or not, cross-link network or continuous film, various film thicknesses, and line widths. For each case, we measured eight devices for calculating the statistical errors. First, the gas-sensing performance of sensors based on 50-nm-thick SnO_2_:NiO network and 50-nm-thick continuous SnO_2_:NiO film are compared in Fig. 6a. It is clear that the ethanol responses of all SnO_2_:NiO film-based sensors are extremely low (< 0.1) whether they were post-annealed or not. This is a common phenomenon for sputtered films due to the close-packed surface structure preventing the exchange of gas molecules. In contrast, the sensing response values of annealed SnO_2_:NiO networks gradually increased to the highest response value with the increase of the operation temperature from 200 to 300 °C. And the responses stayed around 9 at a wide temperature range of 300–375 °C. While further increasing the operation temperature from 375 to 400 °C, the responses decreased rapidly. The significantly increased responses in SnO_2_:NiO networks shows that creating holes is an effective way to enhance the gas-sensing properties of sputtered thin films. Second, annealing is verified to be necessary to activate the networks. During post-annealing at 500 °C, the SnO_2_:NiO network was reorganized to obtain crystallinity and effective surface area. Third, the influence of network thickness on the temperature-dependent sensor responses is also shown in Fig. 6a. The maximum magnitude of sensitivity was obtained for 50-nm-thick networks. This result can probably be explained considering two aspects. On the one hand, step-like surface is more prominent for thicker SnO_2_:NiO networks, which may create more active adsorption sites for gas-sensing. On the other hand, the gain or loss of electrons on the surface of sensing materials due to the adsorbed gas molecules becomes negligible for thicker networks, because most of the conduction paths exist in the internal part of materials. Finally, the influence of plasma etching time on gas-sensing performance is shown in Fig. 6b. The sensor responses at various working temperatures first increases with increasing etching time from 15 to 20 min, and then decreased with a large statistical error for the etching time of 30 min. This large device-to-device deviation can be attributed to the displacement of PS microspheres under constant plasma bombarding, which leads to a disordered cross-linked network. In comparison with the various nanostructured SnO_2_ prepared by other methods in Table 1, the cross-linked SnO_2_/NiO network exhibited comparable sensitivity [19, 23, 47, 49–52]. We also investigated the ethanol sensitivity of other MEMS compatible sensing materials in Table 1, such as DPN deposited Au/SnO_2_ nanocomposites, ZnO nanowires grown on a MEMS microplate, and ZnO tetrapods deposited on a microheater [37, 38, 51]. Apart from the comparable or better sensitivity, there are several other advantages for the cross-linked SnO_2_/NiO networks including high yield, low device-to-device deviation, cheap and simple processing.

**Table 1:** Comparison of the sensing performance between the current work with previously reported results

No.	Sensing element	Method of preparation	Ethanol (ppm)	Op.tem. (°C)	Response^a^	Ref.
1	Nanocomposite core–shell Ag@SnO_2_	Chemical solution route followed by calcination	200	25	2.24	[49]
2	1.00 wt.% La_2_O_3_ and 99.00 wt.% Sb-doped SnO_2_ (Sb-SnO_2_)	Chemical solution route followed by calcination	100	200	16	[23]
3	Pd-doped SnO_2_ hollow microcubes	Two step Chemical solution route and calcination	200	300	90	[50]
4	Nanorods ZnO backbone and SnO_2_ branches	One-step hydrothermal method	100	275	18.1	[47]
5	Au–SnO_2_ nanocomposites	Dip pen nanolithography, deposit on a MEMS platform	1000	375	28	[38]
6	Ni-doped SnO_2_	One-step hydrothermal method	100	260	28.9	[19]
7	ZnO nanowires grown on a CMOS microhotplate	Hydrothermal method	809	400	2	[51]
8	Horseshoe-shaped SnO_2_ with annulus like mesoporous	Self-assembly method	100	225	17.3	[52]
9	ZnO tetrapods	Thermal evaporation and controlled oxidation; deposit on a microheater through a PDMS mask	50	400	30	[37]
10	Cross-linked SnO_2_:NiO network	Magnetic sputtering on etched PS microsphere templates	50	300	9	This work

^a^Response = *R*_a_/*R*_g_ − 1

**Fig. 7:**
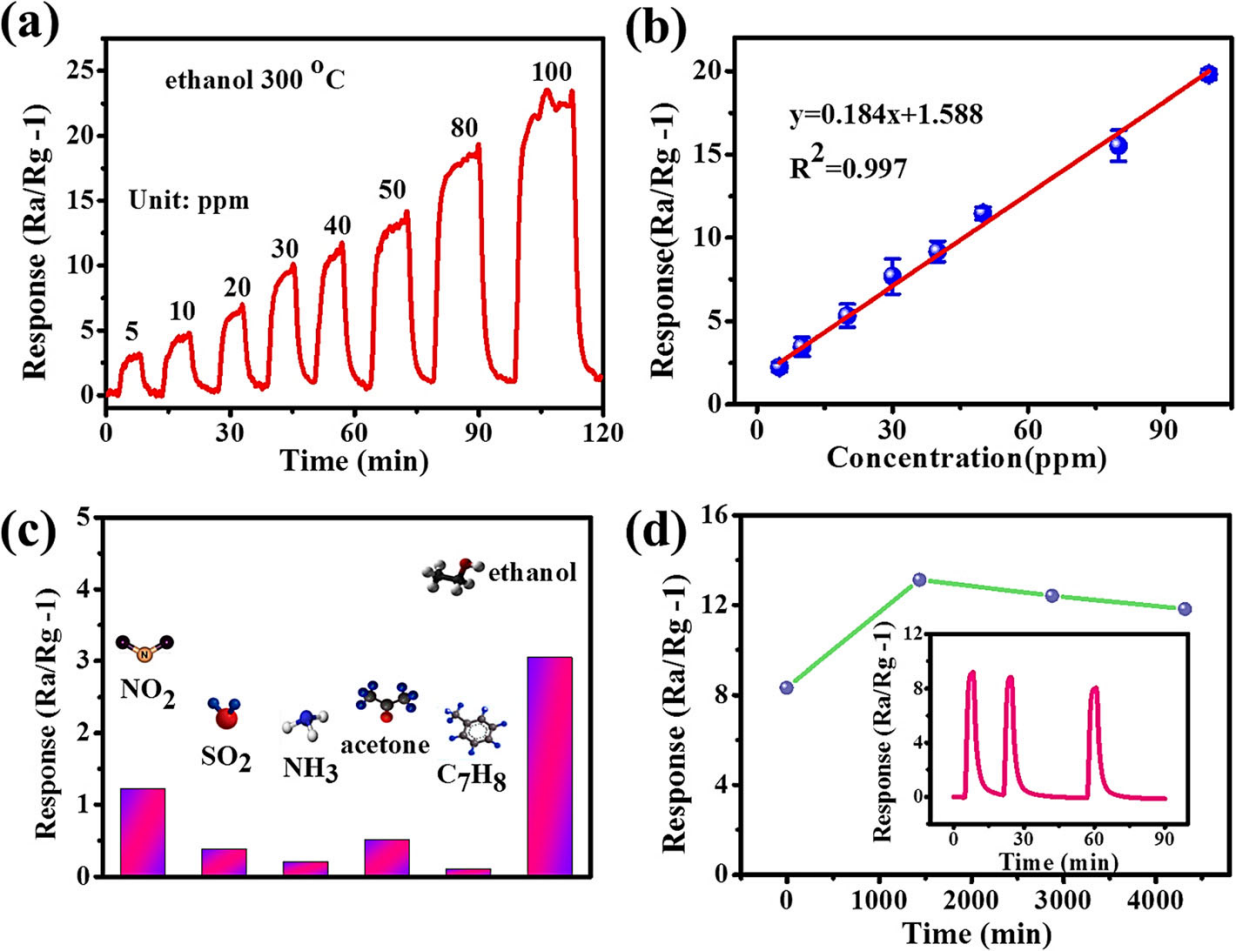
**a** Real-time response curve to different ethanol concentrations at 300 °C. **b** The response linear fitting curve as a function of the ethanol concentration at 300 °C. **c** Gas responses of cross-linked SnO_2_:NiO network to 5 ppm various target gases including NO_2_, SO_2_, NH_3_, acetone, C_7_H_8_, and ethanol. **d** The response stability of a typical SnO_2_/NiO network sensor continuously measured in 3 days to 50 ppm ethanol at 300 °C. The inset figure in (**d**) shows the response-recovery curve of the same sensor measured after 3 days

The typical response and recovery characteristic curve of the network-based sensor to ethanol in the range of 5–100 ppm at 300 °C was shown in Fig. 7a. Obviously, the responses in these curves increased with increasing ethanol concentration. The measured responses are 3.04, 4.58, 6.39, 9.44, 11.00, 13.19, 18.53, and 22.45 for SnO_2_/NiO network corresponding to 5, 10, 20, 30, 40, 50, 80, and 100 ppm, respectively. It can be concluded that a low detection limit of < 5 ppm can be achieved for our network based sensors. However, the measured response and recovery time of network sensor are in the order of minutes, much longer than the nanomaterial-based sensors [53, 54]. Compared with the test system and sensing materials in the reported sensors, we believe that the long response and recovery time in our work can be attributed to the following two reasons. First, we measured the gas-sensing property in a dynamic test system instead of a static test system. The target gas was mixed in a special chamber, and then diffused for a long distance into the quartz tube (50 mm in diameter, 1 m in length) after we open the valve of chamber. It costs more than 1 min for the diffused gas to blow away the synthetic air and reach a stable concentration. Second, the design of cross-linked SnO_2_:NiO networks is based on sputtering films, which show much poor crystallinity and much smaller surface-to-volume ratio. Thus, the exchange of gaseous molecules in such networks is much slower than that in nanostructured sensing materials. Figure 7b shows that the gas sensor shows a linear response to the change of ethanol concentration in the relatively low concentration range (5–100 ppm).

As we all know, selectivity is a key factor for practical applications of a gas sensor. Figure 7c shows the response values of the cross-linked SnO_2_:NiO network upon 5 ppm ethanol and the common interfering gases such as NO_2_, SO_2_, NH_3_, acetone, and toluene at an operating temperature of 300 °C. This result clearly demonstrates that the sensor exhibits better selectivity to ethanol gas. On the one hand, the response of oxidizing gases like NO_2_ mainly depends on the adsorption-desorption of NO_2_ molecules, which is often low efficient at high temperature (> 200 °C). On the other hand, the oxidizing performance for reducing gases depends on their intrinsic reducing ability, which is related to their bond energies. The lower the bond energy is, the easier the reaction occurs. According to the bond energy data of 610.3, 798.9, 548, and 458.8 kJ/mol, respectively for C=C, C=O, S=O, and O-H, it is obvious that O-H bond in ethanol is the weakest [55]. This probably explains the high selectivity to ethanol for our network sensors.

Figure 7d shows the stability of network based sensors. In our test, the sensor was exposed to 50 ppm ethanol for 4 cycles in 72 h at a working temperature of 300 °C. A relatively constant response of around 10 was obtained in the 4-cycle tests. However, the sensor broke down in the fifth cycle because of the electrical degradation under high sensing temperature. Similar problems were reported by Zeng, et al. when they measured the long-term stability of SnO_2_ nanowire sensors at 200 °C [56]. The oxidation of adhesion layer like Ti or Cr leads to a rapidly increased contact resistance, especially in O_2_ atmosphere at high temperature. The inset figure in Fig. 7d shows the response-time curve of the same sensor after redefining gold electrodes three weeks later. The recovery of sensitivity implies the stability of cross-linked SnO_2_:NiO network. High quality of electrical contacts under harsh sensing conditions can be achieved probably by using heavily doped metal oxide and the nitride or carbide of transition metals, which will be investigated in the future work.

### Gas-Sensing Mechanism

**Fig. 8:**
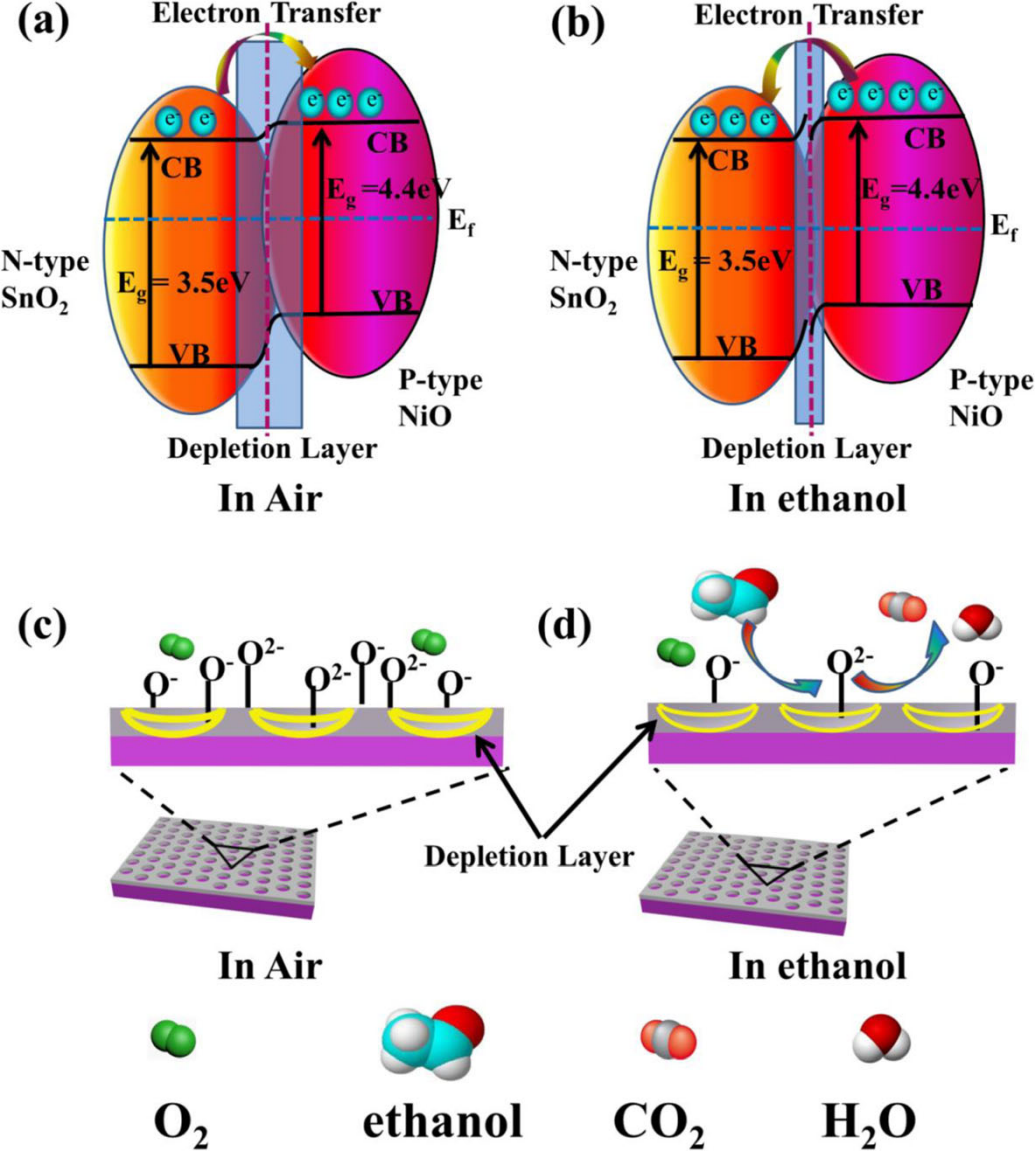
Schematics diagram of gas-sensing mechanism of cross-linked SnO_2_:NiO network. **a**, **b** Schematic diagram of the energy band configurations for SnO_2_:NiO network in air and in ethanol vapor. In the diagram, CB is the conduction band, VB is the valence band, E_g_ is the band gap, E_f_ is the Fermi level, and e^−^ is the charge of an electron. The depletion layers at the SnO_2_/NiO interface are indicated by blue rectangles. **c**, **d** Schematic model showing the sensing mechanism of the SnO_2_:NiO network exposed in air and ethanol, respectively. The yellow lines indicates the wide depletion region in the holes of cross-linked SnO_2_:NiO network

The space-charge layer model has often been applied to explain the detailed change of mobile charge carriers exposed in air and target gases. In SnO_2_:NiO composites, SnO_2_ is a typical n-type MOS with a reported work function of 3.5 eV, and NiO is a p-type material with a work function of 4.4 eV [57, 58]. Thus, p-n heterojunction forms after the post-annealing of SnO_2_:NiO composites, leading to the transfer of electrons from SnO_2_ to NiO in order to get a stable state. A depletion layer appears at the SnO_2_/NiO interface, as indicated by the blue rectangle in Fig. 8a. When exposed in air, the adsorbed oxygen molecules on the surface of SnO_2_ are transformed to oxygen ions (O^−^, O_2_^−^, or O^2−^) by capturing electrons from the conductance band of SnO_2_ network (Eqs. (1)–(4)). The electron-capture process leads to a wide depletion region in SnO_2_, and thus a high resistance state is formed, as shown in Fig. 8c. The yellow bold lines Fig. 8c indicates the wide depletion region in the holes of cross-linked SnO_2_:NiO network. Compared to the pure SnO_2_, the formation of p-n heterojunction leads to a higher sensor resistance in air and a wider depletion region due to the electron transfer from SnO_2_ to NiO.

1 $$ {\mathrm{O}}_2\left(\mathrm{gas}\right)\leftrightarrow {\mathrm{O}}_2\left(\mathrm{ads}\right) $$

2 $$ {\mathrm{O}}_2\left(\mathrm{ads}\right)+{\mathrm{e}}^{-}\leftrightarrow {{\mathrm{O}}_2}^{-} $$

3 $$ {{\mathrm{O}}_2}^{-}+{\mathrm{e}}^{-}\leftrightarrow {2\mathrm{O}}^{-} $$

4 $$ {\mathrm{O}}^{-}\left(\mathrm{ads}\right)+{\mathrm{e}}^{-}\leftrightarrow {\mathrm{O}}^{2-}\left(\mathrm{ads}\right) $$

When the SnO_2_:NiO network sensors are exposed to alcohol vapors (reducing gases), the alcohol molecules adsorbed on the surfaces of SnO_2_ react with the chemisorbed oxygen ions forming CO_2_ and H_2_O, according to Eq. (5) and Eq. (6). The release of free electrons back into SnO_2_ leads to a narrow depletion region in Fig. 8d and a low resistance state. Electrons transfer from NiO back to SnO_2_ in Fig. 8b to get a new uniform Fermi level, because the electron concentration is lower in SnO_2_ than that at the initial state. This transfer of electrons leads to additional conduction paths and a lower resistance state, which probably explains the role of p-n heterojunction in enhancing the gas-sensing performance.

5 $$ {\mathrm{C}}_2{\mathrm{H}}_5\mathrm{OH}\left(\mathrm{ads}\right)+{6\mathrm{O}}^{-}\left(\mathrm{ads}\right)\to {2\mathrm{CO}}_2\left(\mathrm{gas}\right)+{3\mathrm{H}}_2\mathrm{O}\left(\mathrm{gas}\right)+{6\mathrm{e}}^{-} $$

6 $$ {\mathrm{C}}_2{\mathrm{H}}_5\mathrm{OH}\left(\mathrm{ads}\right)+{6\mathrm{O}}^{2-}\left(\mathrm{ads}\right)\to {2\mathrm{CO}}_2\left(\mathrm{gas}\right)+{3\mathrm{H}}_2\mathrm{O}\left(\mathrm{gas}\right)+12{\mathrm{e}}^{-} $$

The creation of steps in sputtered SnO_2_:NiO thin films is proved a key factor to achieve high response, which is positively attributed to the enhanced surface adsorption. On the one hand, the surface of SnO_2_:NiO network is less compact compared with the continuous SnO_2_:NiO film, facilitating the adsorption of gas molecules. The cross-linked SnO_2_:NiO network is composed of interconnecting nanowires. Additional nanostructures like nanocracks appear in these nanowires due to the release of tensile stress in the post-annealing process, which can be demonstrated by the contrast of light and dark in the nanowires in Fig. 3h. On the other hand, sensing area rich of the stepped and kinked crystal surfaces should tend to adsorb more gaseous molecules than those on the other area, because a lower enthalpy of the adsorbed phase exists when a gaseous molecule is adsorbed on such structure. According to thermodynamical theory, the correlation between the changes in Gibbs free energy (G), entropy (S), and enthalpy (H) follow the equation ΔG = ΔH-TΔS [9]. In the process of gas adsorption, Gibbs free energy decreases. It is clear that a lower enthalpy of the adsorbed phase (H_a_) indicates a larger ΔG and more adsorbed gaseous molecules. Considering the creation of nanostructures and the steps in cross-linked network, the senor response of SnO_2_:NiO network is 45-fold higher than that of sputtered continuous SnO_2_:NiO film.

## Conclusion

Cross-linked SnO_2_:NiO networks were successfully fabricated via MEMS compatible self-assembly and template sputtering techniques. The structural parameters of PS microspheres template were controlled to achieve various line widths of interconnecting nanowires in SnO_2_:NiO networks. Gas sensing measurements indicated that the SnO_2_:NiO network sensors were highly sensitive to ethanol. For the optimum structure, SnO_2_:NiO network with plasma etching time of 20 min, the response to 50 ppm ethanol at 300 °C was 9, 45-fold that of continuous SnO_2_:NiO thin film. A linear dependence of the response on the ethanol concentration in the range of 5–100 ppm was observed. The SnO_2_:NiO network showed only minor sensitivity to NO_2_ (1.2 to 5 ppm NO_2_) and even lower sensitivity to other interfering gases. Despite of the electrical degradation of electrodes after continuously operated for 72 h at 300 °C, the SnO_2_:NiO sensing network showed long-term stability of over 3 weeks. The enhanced ethanol sensing performance due to the creation of steps in SnO_2_:NiO network results from an less compact structure and increased adsorption sites.

## Data Availability

The authors declare that the materials, data, and associated protocols are available to the readers, and all the data used for the analysis are included in this article.

## Abbreviations

ALD: Atomic layer deposition
CTAB: Cetyltrimethyl ammonium bromide
CVD: Chemical vapor deposition
DPN: Dip pen nanolithography
MEMS: Microelectrical mechanical system
MOS: Semiconducting metal oxides
PS: Polystyrene
PVD: Physical vapor deposition
SAXS: Small-angle X-ray scattering
SEM: Scanning electron microscope
VOC: Volatile organic compound
XPS: X-ray photoelectron spectroscopy

## References

[1] Wang XX, Ugur A, Goktas H, Chen N, Wang MH, Lachman N, Kalfon-Cohen E, Fang WJ, Wardle BL, Gleasont KK (2016). Room temperature resistive volatile organic compound sensing materials based on a hybrid structure of vertically aligned carbon nanotubes and conformal oCVD/iCVD polymer coatings. Acs Sensors.

[2] Song LF, Luo LQ, Xi Y, Song JJ, Wang Y, Yang LP, Wang AQ, Chen YF, Han N, Wang FY (2019). Reduced graphene oxide-coated Si nanowires for highly sensitive and selective detection of indoor formaldehyde. Nanoscale Res Lett.

[3] Cho SY, Koh HJ, Yoo HW, Kim JS, Jung HT (2017). Tunable volatile-organic-compound sensor by using au nanoparticle incorporation on MoS2. Acs Sensors.

[4] Schnabel R, Fijten R, Smolinska A, Dallinga J, Boumans ML, Stobberingh E, Boots A, Roekaerts P, Bergmans D, van Schooten FJ (2015). Analysis of volatile organic compounds in exhaled breath to diagnose ventilator-associated pneumonia. Sci Rep.

[5] Zhu HB, She JY, Zhou ML, Fan XD (2019). Rapid and sensitive detection of formaldehyde using portable 2-dimensional gas chromatography equipped with photoionization detectors. Sensors Actuators B-Chemical.

[6] Degler D, Rank S, Muller S, de Carvalho HWP, Grunwaldt JD, Weimar U, Barsan N (2016). Gold-loaded tin dioxide gas sensing materials: mechanistic insights and the role of gold dispersion. Acs Sensors.

[7] Wang C, Cui XB, Liu JY, Zhou X, Cheng XY, Sun P, Hu XL, Li XW, Zheng J, Lu GY (2016). Design of superior ethanol gas sensor based on Al-doped NiO nanorod-flowers. Acs Sensors.

[8] Zhang C, Luo YF, Xu JQ, Debliquy M (2019). Room temperature conductive type metal oxide semiconductor gas sensors for NO2 detection. Sensors Actuators a-Physical.

[9] Xu SP, Xu Y, Zhao HP, Xu R, Lei Y (2018). Sensitive gas-sensing by creating adsorption active sites: coating an SnO2 layer on triangle arrays. ACS Appl Mater Interfaces.

[10] Gu FB, Wang HT, Han DM, Wang ZH (2017). Enhancing the sensing performance of SnO2 inverse opal thin films by in and au doping. Sensors Actuators B-Chemical.

[11] Shankar P, Rayappan JBB (2017). Monomer: design of ZnO nanostructures (Nanobush and nanowire) and their room-temperature ethanol vapor sensing signatures. ACS Appl Mater Interfaces.

[12] Xiao L, Xu SR, Yu G, Liu ST (2018). Efficient hierarchical mixed Pd/SnO2 porous architecture deposited microheater for low power ethanol gas sensor. Sensors Actuators B-Chemical.

[13] Kim SY, Kim J, Cheong WH, Lee IJ, Lee H, Im HG, Kong H, Bae BS, Park JU (2018). Alcohol gas sensors capable of wireless detection using In2O3/Pt nanoparticles and Ag nanowires. Sensors Actuators B-Chemical.

[14] Xin X, Zhang JN, Chen CJ, Li G, Qin J, Yang ZB, Lu HB, Gao JZ, Wang CL, He Z (2019). UV-activated porous Zn2SnO4 nanofibers for selective ethanol sensing at low temperatures. J Alloys Compd.

[15] Wang Q, Bai JL, Huang BY, Hu Q, Cheng X, Li JP, Xie EQ, Wang YR, Pan XJ (2019). Design of NiCo2O4@SnO2 heterostructure nanofiber and their low temperature ethanol sensing properties. J Alloys Compd.

[16] Xu Q, Zhang ZC, Song XP, Yuan S, Qiu ZW, Xu HY, Cao BQ (2017). Improving the triethylamine sensing performance based on debye length: a case study on alpha-Fe2O3@NiO(CuO) core-shell nanorods sensor working at near room-temperature. Sensors Actuators B-Chemical.

[17] Zhong WW, Shen SJ, He M, Wang D, Wang ZP, Lin ZP, Tu WG, Yu JG (2019). The pulsed laser-induced Schottky junction via in-situ forming Cd clusters on CdS surfaces toward efficient visible light-driven photocatalytic hydrogen evolution. Appl Catalysis B-Environmental.

[18] Zhong WenWu, Huang Jingdong, Liang Shuquan, Liu Jun, Li Yejing, Cai Gemei, Jiang Yong, Liu Jun (2019). New Prelithiated V2O5 Superstructure for Lithium-Ion Batteries with Long Cycle Life and High Power. ACS Energy Letters.

[19] Li Z, Yi JX (2017). Enhanced ethanol sensing of Ni-doped SnO2 hollow spheres synthesized by a one-pot hydrothermal method. Sensors Actuators B-Chemical.

[20] Sun P, Wang C, Liu JY, Zhou X, Li XW, Hu XL, Lu GY (2015). Hierarchical assembly of alpha-Fe2O3 Nanosheets on SnO2 hollow nanospheres with enhanced ethanol sensing properties. ACS Appl Mater Interfaces.

[21] Guo J, Zhang J, Gong H, Ju D, Cao B (2016). Au nanoparticle-functionalized 3D SnO2 microstructures for high performance gas sensor. Sensors Actuators B-Chemical.

[22] Kou XY, Wang C, Ding MD, Feng CH, Li X, Ma J, Zhang H, Sun YF, Lu GY (2016). Synthesis of co-doped SnO2 nanofibers and their enhanced gas-sensing properties. Sensors Actuators B-Chemical.

[23] Wang MY, Zhu LF, Zhang CY, Gai GS, Ji XW, Li BH, Yao YW (2016). Lanthanum oxide @ antimony-doped tin oxide with high gas sensitivity and selectivity towards ethanol vapor. Sensors Actuators B-Chemical.

[24] Yang L, Zhou X, Song L, Wang Y, Wu X, Han N, Chen Y (2018). Noble metal/tin dioxide hierarchical hollow spheres for low-concentration breath methane sensing. ACS Appl Nano Mater.

[25] Wang Y, Liu CY, Wang Z, Song ZW, Zhou XY, Han N, Chen YF (2019). Sputtered SnO2:NiO thin films on self-assembled Au nanoparticle arrays for MEMS compatible NO2 gas sensors. Sensors Actuators B-Chemical.

[26] Ke MT, Lee MT, Lee CY, Fu LM (2009). A MEMS-based benzene gas sensor with a self-heating WO3 sensing layer. Sensors.

[27] Gurusamy JT, Putrino G, Jeffery RD, Silva K, Martyniuk M, Keating A, Faraone L (2019). MEMS based hydrogen sensing with parts-per-billion resolution. Sensors Actuators B-Chemical.

[28] Vasiliev AA, Pisliakov AV, Sokolov AV, Samotaev NN, Soloviev SA, Oblov K, Guarnieri V, Lorenzelli L, Brunelli J, Maglione A, Lipilin AS, Mozalev A, Legin AV (2016). Non-silicon MEMS platforms for gas sensors. Sensors Actuators B-Chemical.

[29] Kang JG, Park JS, Lee HJ (2017). Pt-doped SnO2 thin film based micro gas sensors with high selectivity to toluene and HCHO. Sensors Actuators B-Chemical.

[30] Liu ZF, Yamazaki T, Shen Y, Kikuta T, Nakatani N (2007). Influence of annealing on microstructure and NO2-sensing properties of sputtered WO3 thin films. Sensors Actuators B-Chemical.

[31] Sharma A, Tomar M, Gupta V (2011). SnO(2) thin film sensor with enhanced response for NO(2) gas at lower temperatures. Sensors Actuators B-Chemical.

[32] Zhao X, Shi W, Mu H, Xie H, Liu F (2016). Templated bicontinuous tin oxide thin film fabrication and the NO2 gas sensing. J Alloys Compd.

[33] Fu K, Chen ST, Zhao J, Willis BG (2016). Dielectrophoretic assembly of gold nanoparticles in nanoscale junctions for rapid, miniature chemiresistor vapor sensors. Acs Sensors.

[34] Jalal AH, Alam F, Roychoudhury S, Umasankar Y, Pala N, Bhansali S (2018). Prospects and challenges of volatile organic compound sensors in human healthcare. Acs Sensors.

[35] Guntner AT, Koren V, Chikkadi K, Righettoni M, Pratsinis SE (2016). E-nose sensing of low-ppb formaldehyde in gas mixtures at high relative humidity for breath screening of lung cancer?. Acs Sensors.

[36] Lee HK, Moon SE, Choi NJ, Yang WS, Kim J (2012). Fabrication of a HCHO gas sensor based on a MEMS heater and inkjet printing. J Korean Phys Soc.

[37] Marasso SL, Tommasi A, Perrone D, Cocuzza M, Mosca R, Villani M, Zappettini A, Calestani D (2016). A new method to integrate ZnO nano-tetrapods on MEMS micro-hotplates for large scale gas sensor production. Nanotechnology.

[38] Santra S, Sinha AK, De Luca A, Ali SZ, Udrea F, Guha PK, Ray SK, Gardner JW (2016). Mask-less deposition of Au-SnO2 nanocomposites on CMOS MEMS platform for ethanol detection. Nanotechnology.

[39] Bittencourt C, Llobet E, Ivanov P, Correig X, Vilanova X, Brezmes J, Hubalek J, Malysz K, Pireaux JJ, Calderer J (2004). Influence of the doping method on the sensitivity of Pt-doped screen-printed SnO2 sensors. Sensors Actuators B-Chemical.

[40] Xu SP, Sun FQ, Gu FL, Zuo YB, Zhang LH, Fan CF, Yang SM, Li WS (2014). Photochemistry-based method for the fabrication of SnO2 monolayer ordered porous films with size-tunable surface pores for direct application in resistive-type gas sensor. ACS Appl Mater Interfaces.

[41] Gurlo A (2011). Nanosensors: towards morphological control of gas sensing activity. SnO2, In2O3, ZnO and WO3 case studies. Nanoscale.

[42] Shim Y-S, Moon HG, Kim DH, Zhang L, Yoon S-J, Yoon YS, Kang C-Y, Jang HW (2013). Au-decorated WO3 cross-linked nanodomes for ultrahigh sensitive and selective sensing of NO2 and C2H5OH. RSC Adv.

[43] Girija KG, Somasundaram K, Topkar A, Vatsa RK (2016). Highly selective H2S gas sensor based on cu-doped ZnO nanocrystalline films deposited by RF magnetron sputtering of powder target. J Alloys Compd.

[44] Behera B, Chandra S (2016). An innovative gas sensor incorporating ZnO-CuO nanoflakes in planar MEMS technology. Sensors Actuators B-Chemical.

[45] Fang JB, Zhu YP, Wu DJ, Zhang C, Xu SH, Xiong DY, Yang PX, Wang LW, Chu PK (2017). Gas sensing properties of NiO/SnO2 heterojunction thin film. Sensors Actuators B-Chemical.

[46] Kaur M, Dadhich BK, Singh R, Ganapathi K, Bagwaiya T, Bhattacharya S, Debnath AK, Muthe KP, Gadkari SC (2017). RF sputtered SnO2: NiO thin films as sub-ppm H2S sensor operable at room temperature. Sensors Actuators B-Chemical.

[47] Yang XL, Zhang SF, Yu Q, Zhao LP, Sun P, Wang TS, Liu FM, Yan X, Gao Y, Liang XS, Zhang SM, Lu GY (2019). One step synthesis of branched SnO2/ZnO heterostructures and their enhanced gas-sensing properties. Sensors Actuators B-Chemical.

[48] Li N, Fan Y, Shi Y, Xiang Q, Wang XH, Xu JQ (2019). A low temperature formaldehyde gas sensor based on hierarchical SnO/SnO2 nano-flowers assembled from ultrathin nanosheets: synthesis, sensing performance and mechanism. Sensors Actuators B-Chemical.

[49] Wu RJ, Lin DJ, Yu MR, Chen MH, Lai HF (2013). Ag@SnO2 core-shell material for use in fast-response ethanol sensor at room operating temperature. Sensors Actuators B-Chemical.

[50] Xiao L, Shu SM, Liu ST (2015). A facile synthesis of Pd-doped SnO2 hollow microcubes with enhanced sensing performance. Sensors Actuators B-Chemical.

[51] Santra S, Guha PK, Ali SZ, Hiralal P, Unalan HE, Covington JA, Amaratunga GAJ, Milne WI, Gardner JW, Udrea F (2010). ZnO nanowires grown on SOI CMOS substrate for ethanol sensing. Sensors Actuators B-Chemical.

[52] Wang YL, Liu C, Wang L, Liu J, Zhang B, Gao Y, Sun P, Sun YF, Zhang T, Lu GY (2017). Horseshoe-shaped SnO2 with annulus-like mesoporous for ethanol gas sensing application. Sensors Actuators B-Chemical.

[53] Wan KC, Wang D, Wang F, Li HJ, Xu JC, Wang XY, Yang JH (2019). Hierarchical In2O3@SnO2 core-shell nanofiber for high efficiency formaldehyde detection. ACS Appl Mater Interfaces.

[54] Zhang Y, Duan ZH, Zou HF, Ma M (2018). Drawn a facile sensor: a fast response humidity sensor based on pencil-trace. Sensors Actuators B-Chemical.

[55] Miller AP (1941). Lange's handbook of chemistry, 11th Ed. Nav Eng J.

[56] Zeng H, Takahashi T, Kanai M, Zhang GZ, He Y, Nagashima K, Yanagida T (2017). Long-term stability of oxide nanowire sensors via heavily doped oxide contact. Acs Sensors.

[57] Yan S, Xue JZ, Wu QS (2018). Synchronous synthesis and sensing performance of alpha-Fe2O3/SnO2 nanofiber heterostructures for conductometric C2H5OH detection. Sensors Actuators B-Chemical.

[58] Hu L, Peng J, Wang WW, Xia Z, Yuan JY, Lu JL, Huang XD, Ma WL, Song HB, Chen W, Cheng YB, Tang J (2014). Sequential deposition of CH3NH3PbI3 on planar NiO film for efficient planar Perovskite solar cells. Acs Photonics.

